# Comparison of long-term benefits of organ-preserving pancreatectomy techniques for benign or low-grade malignant tumors at the pancreatic head

**DOI:** 10.1097/MD.0000000000009420

**Published:** 2017-12-22

**Authors:** Yatong Li, Wenming Wu, Taiping Zhang, Quan Liao, Yupei Zhao, Menghua Dai

**Affiliations:** Department of General Surgery, Peking Union Medical College Hospital, Beijing, China.

**Keywords:** organ-preserving pancreatectomy, pancreatic head resection with segmental duodenectomy, pancreatic head, pancreatoduodenectomy, pylorus-preserving pancreatoduodenectomy

## Abstract

The aim of this article was to investigate and emphasize the clinical benefits of organ-preserving surgeries by comparing the pancreatic head resection with segmental duodenectomy (PHRSD), pylorus-preserving pancreatoduodenectomy (PPPD), and classic pancreatoduodenectomy (PD).

A retrospective analysis of PHRSD (20 patients), PPPD (42 patients), and PD (92 patients) with benign lesions, low-grade malignancies, or early-stage carcinomas at the pancreatic head was performed since 2008. The intraoperative and postoperative courses and a long-term statuses were compared.

The overall average age of the patients in 3 groups was 48.82 years old (range 12–76). The mean operative time and the blood loss were significantly less in the PHRSD and PPPD groups than that in the PD group (*P* < .05), but there were no differences between the PHRSD and PPPD groups. The possibilities of postoperative complications were equivalent in all 3 groups. During an average follow-up time of 61.1 months, there were no recurrence or distant metastasis happened. Patients in the PHRSD and PPPD groups had a better long-term nutritional status because they had less body weight loss (*P* < .01), and suffered less from long-term diarrhea (*P* < .001) than that in the PD group. However, the results in the PPPD group seemed to be better than that in the PHRSD group.

PHRSD and PPPD are ideal procedures of organ-preserving pancreatectomy to fulfill the curative goals of benign lesions, low-grade malignancies, or early-stage carcinomas at the pancreatic head. It was proved to be operative safe and could bring patients with a better nutritional status and quality of life after surgery. However, PHRSD was more difficult with no better long-term benefits than PPPD, which asked a comprehensive consideration when made the surgical choice.

## 1 Introduction

**Figure 1:**
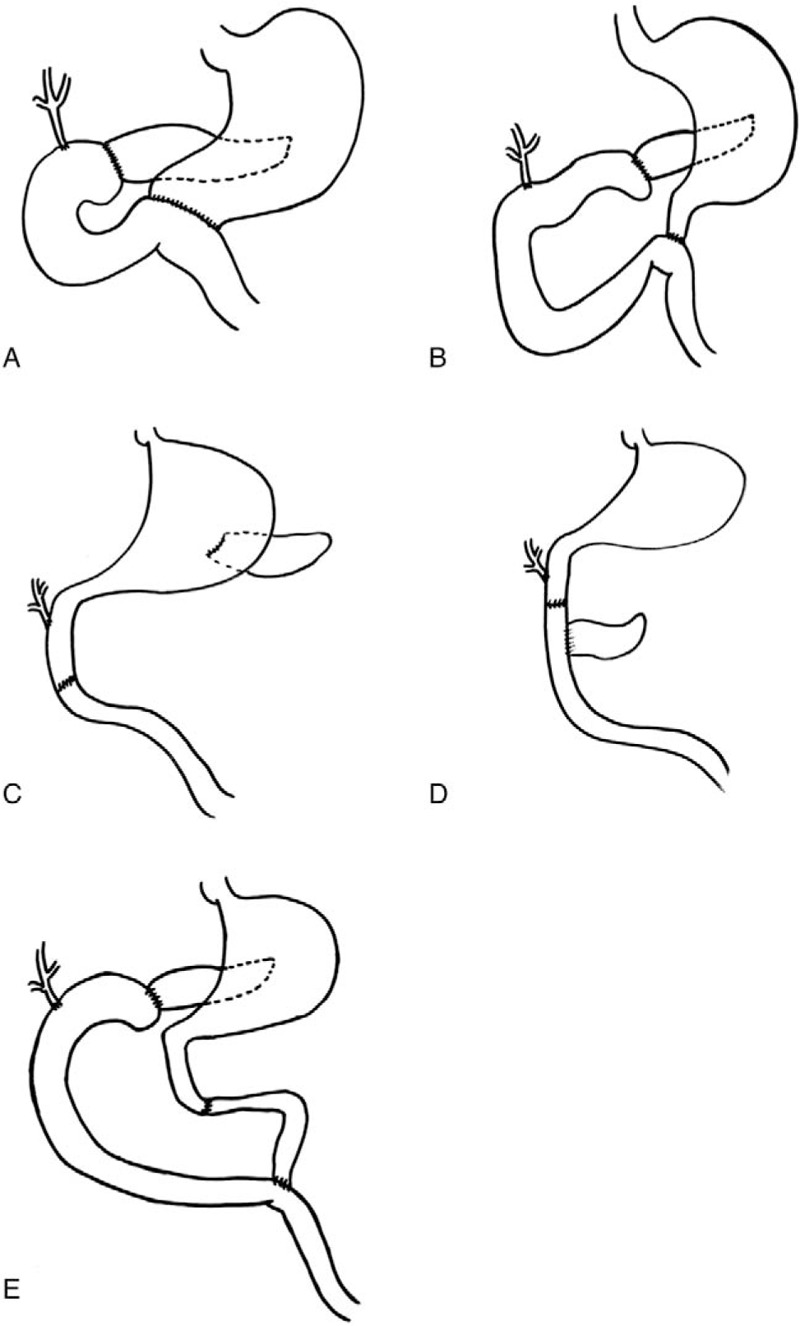
The surgical schematic diagram of PD, PPPD, and PHRSD procedures. (A) The classic PD procedure; (B) the PPPD procedure; (C) the PHRSD procedure with pancreatogastrostomy; (D) the PHRSD procedure with pancreatojejunostomy at the remnant of the duodenum; (E) the PHRSD procedure with pancreatojejunostomy at the jejunum, which was used in our study.

For many years, the classic Whipple procedure (pancreatoduodenectomy, PD) was the standard technique for treatment of any lesions at the pancreatic head.^[1]^ Pylorus-preserving PD (PPPD) subsequently became the principal organ-preserving operation for benign lesions, low-grade malignancies, and early-stage carcinomas at the head of the pancreas (Fig. 1).^[2–5]^ Another organ-preserving procedure, pancreatic head resection with segmental duodenectomy (PHRSD), was first reported in 1994.^[6,7]^ In this operation, only a 3 to 4 cm segment of the duodenum is resected (Fig. 1). As a result, PHRSD preserves important enzymes such as motilin, which is secreted by the duodenum, and has a beneficial effect on intestinal absorption.^[8–10]^

The purpose of organ-preserving procedures such as PPPD and PHRSD is not only to preserve the organ itself but also to improve the postoperative recovery and long-term nutritional status. However, the performance of these procedures and their intended long-term benefits may not be in the patients’ best interests. Because few reports have compared all 3 of these procedures, we performed the present study to identify the differences and advantages of PHRSD, PPPD, and PD.

## 2 Methods

From February, 2008 to November, 2014, 154 patients underwent surgical treatment of benign lesions, low-grade malignancies, or early-stage carcinomas at the head of the pancreas in our hospital. Preoperative enhanced computed tomography and ultrasonography were routinely performed, whereas magnetic resonance imaging, endoscopic ultrasonography, and contrast-enhanced ultrasound were performed as necessary to obtain further information regarding the lesions. If intraoperative frozen pathologic examination of the lesions or resection margins indicated a high-grade malignant neoplasms, PD was performed to ensure the R0 resection and these patients were excluded from the study. Regional lymphadenectomy was performed to ensure benign behavior of the lesions in all 3 procedures. Patients found to have metastatic lymph node were excluded from the study.

The tumor size and surgeon's clinical experience were considered when choosing the operation methods. We advised organ-preserving procedures for patients with smaller tumors, and all patients agreed to these procedures and provided written informed consent. The difficulty of the surgical technology was another important influential factor. Five experienced surgeons performed PD, 3 of them performed PPPD, and 2 performed PHRSD. In total, 20 patients underwent PHRSD, 42 underwent PPPD, and 92 underwent PD. All patients were given the necessary information and provided us with written informed consents.

All the necessary preoperative and postoperative data were collected, and the postoperative complications and long-term nutritional status were retrospectively compared among the PHRSD, PPPD, and PD groups. Diabetes was diagnosed as a fasting blood glucose level of >7.0 mmol/L and a 2-h postmeal blood glucose level of >11.1 mmol/L. Postoperative complications were evaluated according to the modified Clavien-Dindo classification,^[11]^ pancreatic fistula was evaluated based on the International Study Group of Pancreatic Fistula (ISGPF) grading system,^[12]^ and delayed gastric emptying was diagnosed according to the classification of the International Study Group of Pancreatic Surgery (ISGPS).^[13]^ We regarded Clavien grade ≥3, ISGPF grade ≥B, and ISGPS grade ≥B as severe in this study.

Postoperative enhanced computed tomography and ultrasonography were performed every 6 months as follow-up examinations. Blood tests such as measurement of blood routine and serum biochemical indexes were performed during the first month after the operation, every 3 months during the first year, and every 6 months thereafter. The formula used to evaluate changes in the blood indexes was (postoperative value − preoperative value)/preoperative value × 100%. One-way analysis of variance, the χ^2^ test, and the *t* test were used in the statistical analysis, and the software programs used were SPSS 22.0 (IBM Corp., Armonk, NY) and Prism 5 (GraphPad Software, La Jolla, CA). A *P* value of <.05 indicated a statistically significant difference.

## 3 Results

### 3.1 Patients’ demographic and clinical characteristics

The basic characteristics of the 154 patients are summarized in Table 1. Overall, 20 patients underwent PHRSD (6 male, 14 female; average age, 49.5 years; age range, 13–62 years), 42 patients underwent PPPD (23 male, 19 female; average age, 49.8 years; age range, 16–75 years), and 92 patients underwent PD (47 male, 45 female; average age, 48.4 years; age range, 12–76 years). There were no significant differences in these basic characteristics among the 3 groups.

**Table 1:**
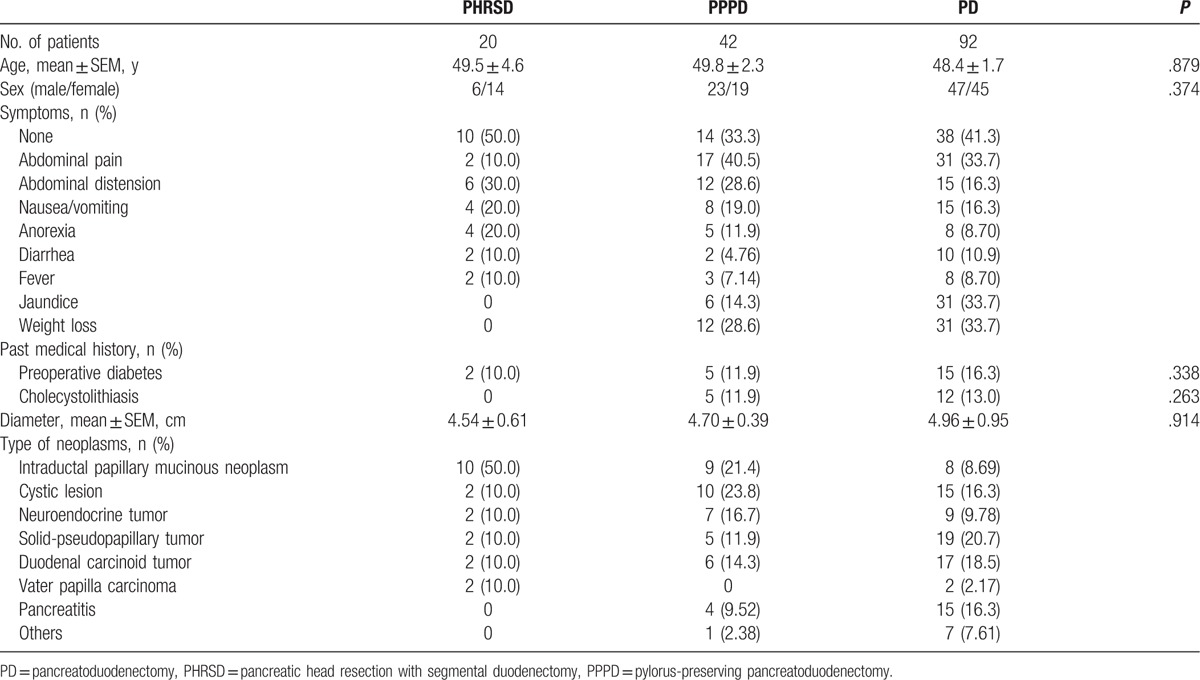
Demographic and clinical characteristics of 154 patients.

The most common preoperative symptoms were abdominal pain, abdominal distension, nausea/vomiting, and anorexia. However, >30% of the patients in each group had no obvious symptoms; these patients’ abnormalities were discovered incidentally during physical examinations. There were no significant differences in the presence of preoperative diabetes or history of gallbladder stones among the 3 groups (Table 1).

The mean diameter of the pancreatic mass in the PHRSD group (4.54 cm) was smaller than that in the PPPD and PD groups (4.70 and 4.96 cm, respectively; Table 1). However, the difference among these diameters was not statistically significant. Although the postoperative pathological diagnoses varied among the patients (intraductal papillary mucinous neoplasms, cystic lesions, pancreatic neuroendocrine tumors, solid pseudopapillary tumors, pancreatitis, and other conditions) (Table 1), all patients had benign lesions, low-grade malignancies, or early-stage carcinomas with low-grade disease progression and no requirement for extensive resection.

### 3.2 Perioperative and postoperative results

No operative or hospital deaths occurred, but the mean operative time and blood loss volume were significantly different among the 3 groups. The statistically significant difference in the length of the hospital stay indicated better recovery after organ-preserving procedures (Table 2).

**Table 2:**
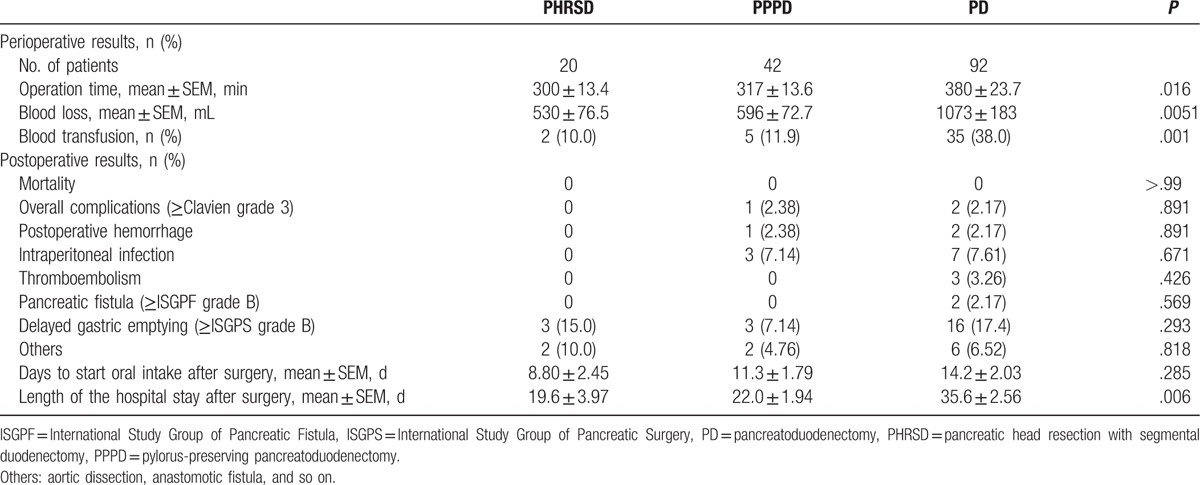
Perioperative status and short-term follow-up.

The mean operative time was significantly shorter in the PHRSD and PPPD groups than in the PD group (300 vs 380 minutes, *P* = .0117; and 317 vs 380 minutes, *P* = .0196, respectively). Likewise, the blood loss volume was significantly lower in the PHRSD and PPPD groups than in the PD group (530 vs 1073 mL, *P* = .0166; and 596 vs 1073 mL, *P* = .0058, respectively). The incidence of perioperative blood transfusion was also significantly lower in the PHRSD and PPPD groups than in the PD group (*P* = .001; Table 2). However, there were no significant differences in any of these 3 indexes between the PHRSD and PPPD groups (*P* = .479, .610, and .828, respectively).

The postoperative hospital was significantly shorter in the PHRSD and PPPD groups than in the PD group (19.6 vs 35.6 days, *P* = .0225; and 22.0 vs 35.6 days, *P* = .0011, respectively), whereas there was still no significant difference between PHRSD and PPPD groups (*P* = .556). With respect to postoperative morbidity, the rate of overall complications (Clavien-Dindo grade ≥3) was not significantly different among the 3 groups, indicating the safety of all 3 procedures (Table 2). We then analyzed the postoperative complications in detail and found no significant differences in the rate of postoperative hemorrhage, intraperitoneal infection, thromboembolism, pancreatic fistula (ISGPF grade ≥B), delayed gastric emptying (ISGPS grade ≥B), or the postoperative duration until starting oral intake (Table 2). These findings indicate that all 3 procedures had the same level of safety.

### 3.3 Long-term pancreatic function and nutritional status

The patients were followed up for a median period of 60 months, during which time no significant differences were observed among the 3 groups. No recurrence, distant metastasis, or death occurred during the follow-up period (Table 3).

**Table 3:**
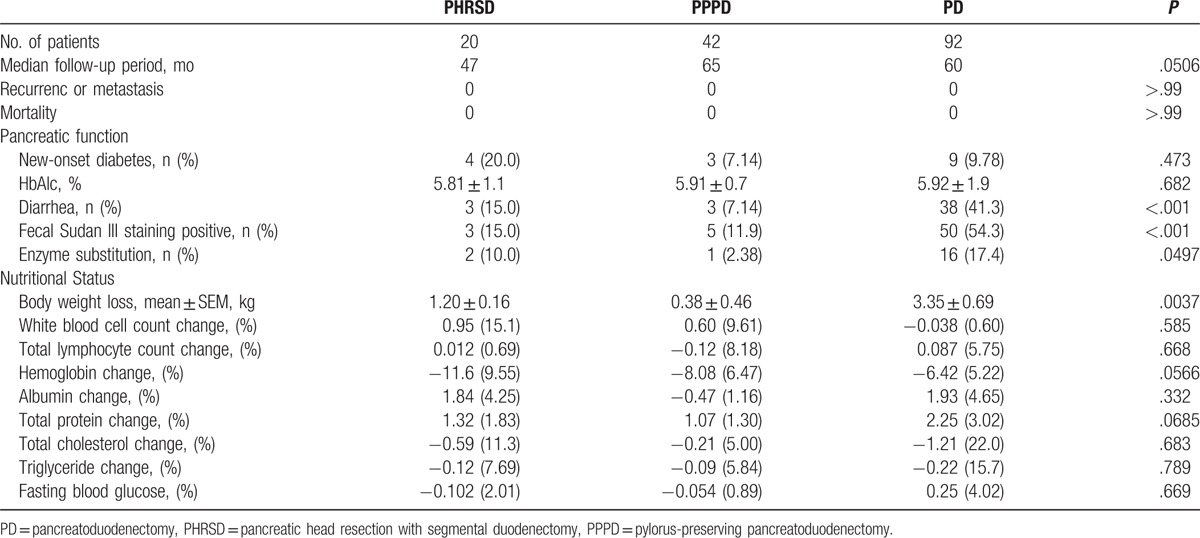
Long-term pancreatic function and nutritional status.

The development of new-onset diabetes, which reflects the pancreatic endocrine function, showed no significant difference among the 3 groups; this condition was evaluated because all 3 operations involve resection of the pancreatic head. Diarrhea, fecal Sudan III staining, and enzyme substitution were important concerns with respect to pancreatic exocrine function, and all 3 were significantly lower in the PHRSD and PPPD groups than in the PD group (*P* < .001, *P* < .001, and *P* = .0497, respectively; Table 3). In addition, the results in the PPPD group seemed to be better than those in the PHRSD group, although the differences were not statistically significant (*P* = .336 and *P* = .269, respectively).

The amount of body weight loss, which was measured 1 year postoperatively, was lower in the PHRSD and PPPD groups than in the PD group (1.20 vs 3.35 kg, *P* = .0421; and .38 vs 3.35 kg, *P* = .0069, respectively), indicating a better long-term nutritional status after the organ-preserving procedures. However, although there was no significant difference in the amount of body weight loss between the PHRSD and PPPD groups (*P* = .0924), the amount of weight loss reflected better recovery after the PPPD procedure. In contrast, blood routine and serum biochemical indexes evaluated 1 year postoperatively seemed to be equivalent in all 3 groups (white blood cell count, total lymphocyte count, hemoglobin level, albumin level, total protein level, total cholesterol level, triglyceride level, and fasting blood glucose level) (Table 3). The reason may be that many more patients in the PD group used enzyme substitution to control diarrhea and increase liquid absorption, resulting in better laboratory values.

## 4 Discussion

PD has long been the standard technique for treatment of any lesions at the pancreatic head. In this procedure, the head of the pancreas, as well as the duodenum, proximal jejunum, gallbladder, distal half of the stomach, and regional lymph nodes are removed because most of these organs share the same arterial blood supply (the gastroduodenal arteries).^[14]^ The mortality rate associated with this procedure in the mid-19th century was extremely high, leading to a strict evaluation of surgeons’ qualifications.^[1,15–18]^ With the development of operative techniques, PD is now very safe. The mortality rate associated with this operation is <3% in high-volume academic medical centers.

Because the operative safety for benign lesions, low-grade malignancies, and early-stage carcinomas generally became guaranteed, more attention was placed on the functional preservation, long-term benefits and better life quality provided by organ-preserving procedures. Thus, PPPD was first reported in 1944 and became popular in subsequent decades.^[19–21]^ As an organ-preserving procedure, the main advantage of this technique is preservation of the pylorus (Fig. 1), which also results in preservation of normal gastric function and emptying.^[22]^ Although whether PPPD is the optimal procedure for every type of carcinomas at the pancreatic head or Vater ampulla remains controversial, it is undisputed that PPPD is safe and a better choice than PD with respect to postoperative complications and long-term life quality in patients with benign lesions, low-grade malignancies, or early-stage carcinomas.^[23–25]^ Our statistical analysis showed that PPPD is beneficial in reducing the operation time, intraoperative blood loss, blood transfusion rate, length of the hospital stay, and long-term body weight loss. In addition, the pancreatic exocrine function is better protected by PPPD than PD.

For further improvement in patient's life quality and long-term nutritional status, PHRSD was first described as another organ-preserving procedure by Nakao et al in the 1990s.^[6,26–28]^ In this procedure, the pancreatic head is completely resected with the lower bile duct and a 3 to 4 cm segment of the duodenum including the major and minor papillae. Three reconstruction methods between the remnant pancreas and the gastrointestinal tract may be used: pancreatogastrostomy, pancreatojejunostomy at the remnant of the duodenum, and pancreatojejunostomy at the jejunum (Fig. 1).^[26,27]^ However, studies have shown no significant differences among these 3 reconstruction methods.^[29–32]^ The decision regarding which method to use is based on the surgeon's preference and the length of the proximal remnant of the duodenum.^[26]^

In the present study, we chose pancreatojejunostomy at the jejunum as our method to reconstruct the digestive system. Thus, we performed 4 anastomosis: one pancreaticojejunostomy, one choledochojejunostomy, one enteral entero-enterostomy, and one duodenal entero-enterostomy (Fig. 1). Nevertheless, the incidence rate of postoperative pancreatic fistula and anastomotic fistula in the PHRSD group was not higher than that in the PPPD or PD groups in our study (Table 2). As a result, the anatomy and function of the duodenum are preserved and the incidences of postoperative complications such as biliary and duodenogastric reflux, duodenal fistula, and delayed gastric emptying are reduced, leading to a better postoperative recovery and long-term benefits. Our study proved these and other benefits of organ-preserving procedures, which have rarely been proven by comparison among all 3 procedures.

However, PHRSD is a more difficult technique and thus takes more time to learn. PHRSD is not a new procedure, but its technical difficulty has limited its popularity.^[6,7,26,27]^ In contrast to PPPD, the duodenal branches of the gastroduodenal artery as well as the anterior inferior pancreatoduodenal artery should be preserved, requiring a careful and highly precise operation.^[26,27]^ Anatomically, the anterior superior pancreatoduodenal artery runs toward the papilla of Vater, and then proceeds to the posterior surface of the pancreas where it joins the anterior inferior pancreatoduodenal artery. The place for the anterior superior pancreatoduodenal artery courses along the posterior side of the pancreas is the mesoduodenum between the third portion of the duodenum and the pancreas.^[6,26,28]^ Thus, this region must be carefully dissected and the blood vessel must be preserved to ensure survival of the duodenum, even though the pancreatic head is resected. If this failed, the duodenum or common bile duct will develop ischemia and necrosis.^[33,34]^

Whether PHRSD is better than PPPD in terms of postoperative recovery and the long-term nutritional status remains controversial. In the present study, we found no significant differences in these indexes between the PHRSD and PPPD groups, which is consistent with some large randomized controlled trials.^[35]^ In addition, considering the incidence of long-term diarrhea, use of enzyme substitution, and amount of body weight loss, the patients in the PPPD group seemed to have a better nutritional status than those in the PHRSD group in our study (Table 3). Thus, we need to evaluate whether such outcomes are worthwhile by performing such a difficult procedure, and the surgical method should be chosen after comprehensive consideration. The main surgical goals are less injury, lower recurrence and metastasis rates, and a better long-term status. Organ-preserving procedures may achieve some but not all of these goals. Some of the intended long-term benefits, which we considered would be provided by some organ-preserving procedures, may be controversial when compared with other procedures, such as PHRSD and PPPD. Precise knowledge and careful operation are mandatory for PHRSD; the risk of necrosis of the remnant duodenum and common bile duct cannot be ignored, and whether this procedure results in better long-term recovery remains controversial. For these reasons, PHRSD has not become a popular procedure. Therefore, as 2 mature and safe procedures of organ-preserving PD, whether PHRSD or PPPD has a better outcome and which is preferred by surgeons worldwide are good questions.

The development of techniques in laparoscopic pancreatic surgery may help to answer these questions. Laparoscopic surgery has the advantages of a minimal incision, less intraoperative blood loss, fewer postoperative complications associated with the surgical wound, and a shorter hospital stay.^[36–38]^ The first laparoscopic PD procedure was reported by Gagner and Pomp in 1994, and their procedure has since been verified and developed worldwide.^[37,39,40]^ At the same time, robotic surgery (da Vinci surgery) has become increasingly more popular.^[41]^ These 2 kinds of minimally invasive surgery could provide a more optimal surgical field with a sufficient operative space, allowing for an easier and more secure PHRSD procedure.

The present study has 2 main limitations. First, the total number of patients was limited. In our hospital, only 154 patients had benign lesions, low-grade malignancies, or early-stage carcinomas at the pancreatic head, and only 20 of them underwent PHRSD. In addition to the surgical difficulties and lack of surgeons’ experience with PHRSD, another probable reason for the low number of patients who underwent this procedure is that some patients were suspected to have a high-grade malignant lesion at the pancreatic head before the surgery. Thus, PD was performed to ensure the clearest resection, although the final pathological diagnosis was a benign or low-grade malignant mass. Therefore, it is important to improve the quality and accuracy of preoperative examinations. Second, the overall follow-up period in our study was not very long. Consequently, more studies with more patients and a longer follow-up period are necessary for further comparison of PHRSD and PPPD.

## 5 Conclusions

The findings in our study provide valuable insights regarding the intraoperative, postoperative, and long-term advantages of organ-preserving operations. PHRSD and PPPD are ideal procedures for patients with benign lesions, low-grade malignant tumors, and early-stage carcinomas at the pancreatic head (e.g., intraductal papillary mucinous neoplasms, other cystic lesions, pancreatic neuroendocrine tumors, solid-pseudopapillary tumors, and pancreatitis). Sometimes, the inflammation associated with severe pancreatitis may make it difficult to preserve some arteries such as the gastroduodenal artery, which is necessary in the PHRSD procedure. However, these organ-preserving operations can fulfill the curative goals for most lesions at the pancreatic head. They have been proven safe and can provide patients with a better postoperative nutritional status and life quality than the classic PD procedure. Thus, they should be advocated more.

On the contrary, the performance of these procedures and their intended long-term benefits may not be in the patients’ best interests. In the PHRSD procedure, a longer portion of the duodenum is preserved and a better long-term status is expected, but our results did not fully support these conclusions. In contrast, some indexes showed a better trend in the PPPD than PHRSD group, implying a better long-term outcome after PPPD. Moreover, PHRSD is a much more difficult procedure than PD and has a much higher risk of organ necrosis. Accordingly, the choice of organ-preserving procedures must be comprehensively evaluated.
